# Sarcopenia: Influence of Regional Skeletal Muscle Cutoff Points and Fat-Free Mass in Older Mexican People—A Pilot Study

**DOI:** 10.1155/2020/8037503

**Published:** 2020-05-31

**Authors:** Heliodoro Alemán-Mateo, Miriam T. López-Teros, Roxana E. Ruiz-Valenzuela, Maribel Ramírez-Torres, René Urquidez-Romero

**Affiliations:** ^1^Coordinación de Nutrición, Centro de Investigación en Alimentación y Desarrollo, A.C. Carretera Gustavo Enrique Astiazarán Rosas #46, Col. La Victoria C.P. 83304, Hermosillo, Sonora, Mexico; ^2^Departamento de Salud, Universidad Iberoamericana, Ciudad de México, Prolongación Paseo de Reforma 880, Lomas de Santa Fe, C.P. 01219, Ciudad de México, Distrito Federal, Mexico; ^3^Departamento de Salud, Universidad Iberoamericana, Ciudad de México-Tijuana. Av. Centro Universitario 2501, Playas de Tijuana, C.P. 22500, Tijuana, Baja California, Mexico; ^4^Coordinación de Nutrición Humana, Universidad Estatal de Sonora, Av. Ley Federal del Trabajo, Col. Apolo, C.P. 83100, Hermosillo, Sonora, Mexico; ^5^Departamento de Ciencias de la Salud, Instituto de Ciencias Biomédicas, Universidad Autónoma de Ciudad Juárez, Ave. Plutarco Elías Calles #1210, Col. Fovissste Chamizal. Ciudad Juárez, Chihuahua, Mexico

## Abstract

**Background:**

Variation in the prevalence of sarcopenia is related to the skeletal muscle index cutoff points applied. The objective of this pilot study was to examine the recruitment process for testing different sarcopenia definitions (ASMI cutoffs) in older Mexican adults. It explored whether the prevalence of sarcopenia decreased by applying ethnic- and gender-specific, DXA-derived appendicular skeletal muscle index (ASMI)-cutoff points in the definitions, as well as some associated factors in a sample of community-dwelling older Mexican people.

**Methods:**

This is a pilot feasibility study that included a convenience sample of 217 community-dwelling older adults. Volunteers underwent DXA measurements and an assessment of functional status based on hand grip strength and physical performance. Six definitions were formed based on the 2010 EWGSOP criteria, but using different cutoff points for each of the three components, including regional cutoff points for ASMI derived from young Mexican adults. Several risk factors for sarcopenia were also assessed.

**Results:**

The prevalence of sarcopenia varied according to the different definitions applied. The lowest level was found with the definition that applied regional ASMI-cutoff points (*p* < 0.01). The sarcopenic older adults had significant lower body weight, fat mass, and fat-free mass (FFM) than the nonsarcopenic subjects. The risk of sarcopenia increased with age and low FFM (*p* < 0.001).

**Conclusion:**

The present study demonstrates the feasibility of the main study, and our data support the notion that using regional ASMI cutoff points resulted in a low prevalence of sarcopenia. Therefore, it is preferable to estimate the prevalence of this condition using ethnic- and gender-specific cutoff points and to explore associated factors such as low FFM.

## 1. Introduction

Despite advances in sarcopenia research and the establishment of diagnostic criteria for some international groups [1–4], there is still no universal consensus as to what criteria and which methods should be used to measure skeletal muscle (SM), and which cutoff points are optimal for diagnosing this condition [5]. Sarcopenia is characterized by the loss of SM, decreased strength, and impaired functional capacity [1, 6]. Therefore, diagnosing sarcopenia involves measuring SM and muscle strength in a reference population of young adults, and in the older adults, at-risk population. In our region and, likely, in others, the unavailability of the infrastructure necessary to quantify SM and its functionality by precise and accurate methods, such as dual-energy X-ray absorptiometry (DXA) and magnetic resonance imaging, limits the capacity to perform sarcopenia diagnoses. This is probably the reason why many countries lack the gender-specific cutoff points needed to define low muscle mass or low muscle strength at the population level.

Today, the prevalence of sarcopenia can only be estimated or, in some cases, diagnosed for specific research or intervention purposes, but not for clinical practice. Despite this limitation, some projects have been conducted in Mexico [7–15] and other Latin American countries [16, 17]. To the best of our knowledge, nine studies related to sarcopenia have been performed in Mexico [7–15]. In all those cases, the 2010 European Working Group on Sarcopenia in Older People's (EWGSOP) criteria [1] were applied, but our review found different figures for the prevalence of sarcopenia. Upon examining the most recent findings on this issue [5, 7, 18–22], it seemed likely that the variation observed in those studies [7–15] was due to the cutoff points for SM applied [5, 7, 18–22], although we did not discount factors such as the age of the study population, settings, and the SM method used to estimate or measure prevalence.

With respect to the variation in the prevalence of sarcopenia, a previous analysis that defined sarcopenia only by low SM concluded that this reflects (1) the diagnostic method and SM cutoff points applied, (2) subjects' characteristics, and (3) the reference population cited [23]. However, more recent findings in which authors applied the 2010 EWGSOP criteria [1] clearly show that high prevalence of sarcopenia corresponded to high SM cutoff points [5, 7, 18–22]. In addition, an effect of ethnicity on skeletal muscle index (SMI) has been reported with young Caucasian adults having higher indices than any other ethnic group [21]. Therefore, it is to be expected that the prevalence of sarcopenia will be lower in older Mexican people if we use indices of regional appendicular skeletal muscle mass (ASMI) of 5.86 kg/m^2^ and 4.72 kg/m^2^ derived from young adult Mexican men and women, respectively, than the prevalence estimated using the ASMI-cutoff points derived from the young adult Caucasian population. This is because the latter have higher ASMI values (7.26 kg/m^2^ and 5.50 kg/m^2^ in men and women, respectively) than Mexicans and other ethnic groups [24, 25].

Significantly, none of the studies published in Mexico [7–15] used DXA-derived ASMI cutoff points from a young adult population to identify older subjects with low muscle mass. On the other hand, sarcopenia is strongly associated with physical disability [26], cognitive dysfunction [27], functional decline [28], loss of quality of life [29], and mortality [30]. Therefore, the early identification of sarcopenia and a better understanding of the factors associated with it will help define strategies and policies to prevent this condition. The primary aim of this pilot study, therefore, was to examine the recruitment process and secondary to explore different sarcopenia definitions (ASMI-cutoffs) in older Mexican adults. We proposed to test whether the figures of sarcopenia prevalence decreased by applying ethnic- and gender-specific DXA-derived SMI-cutoff points in the definition, as well as to identify some associated factors in a sample of community-dwelling older Mexican people.

## 2. Subjects and Methods

This study is based on a nonrandomized sample of 217 of the 223 community-dwelling older adults who met the following specific inclusion and exclusion criteria:

- Men and women ≥60 years old.
- Those who lived in the community for at least five years.
- Apparently healthy by self-report and diseased subjects (type 2 diabetes, hypothyroidism, and high blood pressure) and physically independent subjects.
- Those without pathologies responsible for rapid changes in body composition, such as congestive heart failure, renal and liver failure, cancers, noncontrolled hypo- or hyperthyroidism, type I diabetes, and cognitive dysfunction.
- Those with stable body weight (without weight change >5% during the prior 3 months).
- Those who had difficulty in understanding the instructions given for the procedures were excluded. Also, those whose height or weight was outside the dimensions of the DXA measuring table. Only six volunteers were excluded for one of these reasons.

All volunteers were recruited in the city of Hermosillo, Sonora, Mexico, by phone calls, visits to centers for independent older adults, home visits, and through social networks and flyers. Regarding the recruitment rate, the majority (80–90%) of the older people who meet the abovementioned eligibility criteria agreed to participate and underwent body composition measurements and physical performance tests.

### 2.1. Data Collection

All volunteers underwent DXA body composition measurements and an assessment of functional status based on hand grip strength (HGS) and physical performance tests. Information on their health status (clinical assessment including blood pressure, body weight and height measurements, comorbidities, medication, toxicities, and general health, among other parameters) and demographic characteristics (age, gender, schooling and income levels, marital status, and occupation) was obtained through questionnaires applied during face-to-face interviews. All study procedures were conducted in the Body Composition and Functionality Laboratory at the Coordination of Nutrition of the Research Center for Food and Development, Hermosillo, Sonora, Mexico, from May 2013 to July 2016.

### 2.2. DXA-Appendicular Skeletal Muscle Mass (ASM) and Other Body Composition Determinations

Body composition was measured by densitometry using the Discovery WI (QDR SERIES) densitometer (Hologic, Waltham, USA), as published previously [31]. The ASM measurements were divided by height-squared to obtain the ASMI, km/m^2^. To define low SM, the ASMI-cutoff points previously published were considered [24]. Also, a quintile distribution was performed to determine gender-specific cutoff points based on the lowest quintile value of the ASMI in the total sample of older adults. From the DXA scans, fat-free mass (FFM, kg) represents the sum of bone mineral content (BMC) and total lean tissue measurements. Fat mass (FM) was also obtained from the DXA scans, and then FM, in kg, was divided by height-squared to derive the fat mass index (FMI, kg/m^2^). Height, body weight, body mass index (BMI), and circumferences were also assessed.

### 2.3. Muscle Strength by the HGS Test

Muscle strength assessed by HGS is a key component of the sarcopenia criteria [1]. HGS (kg) was measured using the Takei Smedley dynamometer (Takei Scientific Instruments Co., LTD, Niigata, Japan) according to the manufacturer's recommendations. The highest value measured was used to define low muscle strength. Published cutoff points were applied [1]. The HGS cutoff points were stratified by the BMI, kg/m^2^ and gender (Table 1).

### 2.4. Physical Performance Assessment by the Short Physical Performance Battery (SPPB) and Gait Speed (GS) Tests

Difficulty in performing various movements was assessed by the SPPB, including the standing balance, chair stand, and gait speed tasks. Subjects with scores ≤8 on the SPPB were classified as having low physical performance [1]. GS was considered as an independent variable to identify subjects with low physical performance. GS cutoff points were adjusted by height and gender. Both tests were incorporated into the definitions of sarcopenia (Table 1).

### 2.5. Definitions

Diagnoses of sarcopenia were based on the 2010 EWGSOP criteria [1], but different cutoff points were applied for each one of the three components. Six definitions were formed to conduct the diagnoses. Definition I included the gender-specific DXA-derived ASMI cutoff points of healthy young Mexican adults aged 20–40 years [24]. The HGS and GS cutoff points were based on the quartile distribution of the current study population. The HGS cutoff points were adjusted by BMI and gender, while the GS cutoff points were adjusted by height and gender. The rest of the definitions and their corresponding cutoff points are shown in Table 1.

### 2.6. Presarcopenia

The presarcopenia stage is characterized by low SM but no impact on muscle strength or physical performance [1]. This condition was determined in relation to the different definitions of low ASMI applied (Table 1).

### 2.7. Associated Factors for Sarcopenia

Several well-known associated factors for the three independent components of sarcopenia, or sarcopenia syndrome, were assessed [1, 6, 20, 32]. Therefore, age, gender, schooling (self-reported and recorded as primary, secondary, high school, undergraduate, Master's, and Doctorate), income level to classify socioeconomic status (estimated from total monthly family income) (SES), and health conditions were all recorded and explored as associated factors. The latter included comorbidities, polypharmacy, cognitive dysfunction, physical activity levels (PAL), and smoking and alcohol consumption, all of which were assessed according to the protocol reported previously [31]. Waist circumference (WC) was measured, and low body weight, low FM, and low FFM were explored as markers of undernutrition. These three variables were grouped independently into tertiles: the first tertile of each variable was considered low and a marker of undernutrition, assuming that fat mass and fat-free mass are the ideal parameters for defining malnutrition [33]. Physical dependence was assessed by two methods: activity of daily living (ADL) using the Barthel index [34], and instrumental activities of daily living (IADL) using the Lawton-Brody scale [35].

### 2.8. Statistical Methods

Because this is a pilot study, power and sample size were not calculated. While we recognize that the primary purpose of pilot studies is not to test hypotheses, we took care to include between 10 and 89 subjects in each of the six definitions. Our objective was not to provide appropriate power for hypothesis-testing but, rather, to determine the feasibility of participant recruitment and study design and to identify sarcopenic subjects [36]. To partially achieve these objectives, the figures for the overall prevalence of sarcopenia found by the various definitions were compared using a proportion test. Significant differences between sarcopenic older adults (SOAs) and non-SOA subjects on the various biological and demographic variables were tested by a Student's *t*-test or Chi-squared test, while the main associated factors for sarcopenia were explored by univariate analysis using variables with a *p* value ≤0.2. Next, a multiple logistic regression analysis was run using the multivariate stepwise regression method. The variables with a *p* value ≤0.05 were selected to build the model, which was then evaluated for multiple logistic regression assumptions (i.e., linearity and collinearity). The interaction of all the variables in the models with gender was tested at *p* ≤ 0.1. All analyses were performed using STATA, version 11.0 (Stata Corp, College Station, TX, USA).

## 3. Results

The overall prevalence of sarcopenia estimated using the six definitions varied greatly, with significant differences among all results (Table 2). Definitions I and II generated the lowest prevalence, while definition III (based on the DXA-derived ASMI of a young adult Caucasian population) produced the highest one (*p* < 0.01). Definitions IV, V, and VI indicated similar, moderate prevalence of sarcopenia for the entire sample, though definition IV produced a low prevalence (17.1%). Most of the subjects classified as sarcopenic by definition I had severe sarcopenia (3.2%), though definition III produced the highest prevalence of severe sarcopenia (24.4%). Regarding presarcopenia, overall prevalence depended on the definition applied and ranged from 3.7 to 12.9%.

In order to explore how well definition I classified sarcopenic and nonsarcopenic subjects, we analyzed the behavior of several demographic, anthropometric, body composition, and functional variables (Tables 3). This procedure revealed that the SOAs weighed up to 17.1 kg less than the non-SOA (*p* ≤ 0.05 ) subjects, according to definition I, and had significantly lower mean BMI and fat mass values. They also had less central fat according to the WC. The values of nonadipose tissues, such as FFM and ASM, were lower across all definitions. The mean BMC value was also significantly lower among the SOAs, except for those classified by definition I. As expected, the ASMI was significantly lower across all definitions in the SOAs; however, the lowest ASMI values were found for definitions I and II. HGS was significantly lower in those classified by definitions I, II, III, IV, and V, compared to the non-SOA group. Once again, the sarcopenic subjects identified by definition I had the lowest HGS values. Finally, the mean values from the physical performance assessment by the SPPB and GS tests were lower in the SOAsthan non-SOA subjects.  Table 3 also shows that the former subjects were older (*p* ≤ 0.05), but that none of the other demographic factors explored showed significant between-group differences.

Table 4 shows the comparison of the categories of several markers of undernutrition. Due to their low body weight, fat mass, and FFM, subjects in tertile 1 were most frequent among the SOAs across all definitions (*p* ≤ 0.05). The same results were obtained for PAL, as the sedentary/light activity subjects were more frequent among the SOAs, except for definition IV. In addition, the proportion of subjects with cognitive dysfunction, high blood pressure, osteoporosis, and alcohol consumption differed between groups under some of the definitions applied (see Table 4).

## 4. Discussion

Currently, sarcopenia is considered a disease; however, there is still no universal consensus on its diagnoses [1–4, 37]. Substantial variation in the reported prevalence of sarcopenia is well recognized, and the latest evidence clearly shows that this variation is directly related to the SM cutoff points applied [5, 7, 18–22]. Our data reveal that findings on prevalence were directly influenced by the SM cutoff points used. Specifically, the use of ethnic- and gender-specific ASMI-cutoff points resulted in low prevalence. In contrast, applying nonethnic-specific ASMI-cutoff points generated higher prevalence (Table 2). Our results are supported by other studies [5, 7, 18–22]. Indeed, one recent report found that an increase from 5.45 to 6.68 kg/m^2^ in the total SM index for female out-patients and nursing home residents increased prevalence from 4 to 23% and 9 to 47%, respectively. In men under these same conditions, an increase from 7.25 to 8.87 kg/m^2^ increased prevalence from 1 to 22% and 6 to 41%, respectively [19]. Therefore, it was to be expected that the use of SM cutoff points derived from a population of young adult Caucasians [38], which are higher than the SM cutoff points derived from a young adult Mexican population [24], resulted in a higher prevalence of sarcopenia (Table 2). Our results further confirm that the variation in the cutoff points of GS, SPPB, and HGS did not contribute to the variation, as has been reported elsewhere [19].

Although our sample is not randomized or representative, the present results offer a very clear idea of the importance of avoiding variations in sarcopenia prevalence using regional cutoff points based on the SM measured by DXA in a young reference population. These results indicate that our proposal for a major main study is feasible. However, the overall prevalence of sarcopenia determined by definition I is relatively low—and possibly underestimated—due to the characteristics of the sample, since the older adults who participated were selected in accordance with specific inclusion and exclusion criteria. These limitations can be resolved easily but, more importantly, the results of this pilot study (which included the cutoff points recommended by EWGSOP, 2010 [1]) highlight the need to generate our own cutoff points at the national level due to the evident ethnic and regional differences in body composition, particularly ASMI. Most of the studies done in Mexico have used these criteria and the recommended cutoff points. A possible reanalysis of the prevalence of sarcopenia in older Mexican adults should be considered in light of these results, which are widely supported by several recently published papers [5, 7, 18–22]. Finally, our results are important because the SM cutoff remained within the range of the new EWGSOP criteria [4].

With respect to the associated factors explored herein, Supplementary Tables 1 and 2 show that the age and low FFM categories were the only ones found to be significantly associated with sarcopenia, according to definition I (Supplementary Materials). Some reports sustain that the contributing factors for sarcopenia depend completely on the definitions used [20]; so to maintain congruence with our objectives, we only utilized definition I to look for the association. On this basis, we confirmed that age increases the risk for sarcopenia and, at the same time, found that the risk for sarcopenia increased in subjects with low FFM values (Model 1). This finding may be related to significantly low body weight, BMI, and FFM, as was found in SOAs across all definitions (Table 3). It has been suggested that low BMI is a marker of malnutrition [20, 39], and it is widely accepted that malnutrition can potentiate the onset and progression of sarcopenia [40, 41]. In fact, the most recent GLIM criteria for diagnosing malnutrition include the FFM index cutoff points [42]. Recently, a BMI <20 as a marker of undernutrition was independently associated with sarcopenia [43].

Our pilot study has some additional limitations. First, it involved only subjects ≥60 years who met specific inclusion criteria, which means that we obtained a sample of apparently healthy older adults, some of whom had controlled chronic diseases. Therefore, the prevalence reported herein may be underestimated due to the characteristics of the sample. Second, in terms of hypothesis-testing, the cross-sectional design used is one of the most oft-recommended procedures for estimating prevalence. However, this pilot study explored whether we could detect significance in the figures of prevalence using regional cutoff points for SM in comparison to others. Our results proved that this is completely feasible. With respect to sample size, the number of subjects included had sufficient power to discern statistical differences. In order to determine the sample size for the main trial, the standardized effect size will be required. Finally, our results cannot be generalized because, first, our sample was neither random nor representative and, second, a pilot study is not suitable for testing hypotheses.

## 5. Conclusions

The lowest prevalence was found using definition I, which included regional DXA-ASMI cutoff points, while the highest was estimated by definition III, which was based on DXA-ASMI cutoff points derived from a population of young Caucasian adults. In view of these findings, this pilot study showed the feasibility of the full study, the recruitment process was well accepted, and provided data support the affirmation that regional ASMI cutoff points are preferable due the decreased prevalence of sarcopenia in this sample analyzed and, probably, also for estimating the prevalence of sarcopenia in a more realistic way. Additional studies are highly desirable, especially work focused on morbidity and mortality outcomes associated with sarcopenia (defined using ethnic- and gender-specific cutoff points).

## Figures and Tables

**Table 1:** Six different definitions for diagnosing sarcopenia based on the 2010 EWGSOP criteria.

Men	Women
*Definition I* ^*1,2*^	
ASMI ≤ 5.86 kg/m^2^	ASMI ≤ 4.72 kg/m^2^
HGS-BMI ≤ 24.6; <27 kg	HGS-BMI ≤ 25.1; <15.5 kg
HGS-BMI 24.7–26.3; <32 kg	HGS-BMI 25.2–28.6; <18.5 kg
HGS-BMI 26.4–28.6; <37 kg	HGS-BMI 28.7–31.6; <21.5 kg
HGS-BMI ≥ 28.7; <45 kg	HGS-BMI ≥ 31.7; <25.0 kg
Ht ≤ 169 cm (GS <0.76 m/s)	Ht ≤ 154 cm (GS < 0.74 m/s)
Ht > 169 cm (GS <0.79 m/s)	Ht > 154 cm (GS < 0.79 m/s)

*Definition II* ^*1,3*^	
ASMI ≤ 5.86 kg/m^2^	ASMI ≤ 4.72 kg/m
GS ≤ 0.8 m/s	GS ≤ 0.8 m/s

*Definition III* ^*4*^	
ASMI ≤ 7.26 kg/m^2^	ASMI ≤ 5.50 kg/m^2^
HGS ≤ 30 kg	HGS ≤ 20 kg
GS ≤ 0.8 m/s	GS ≤ 0.8 m/s

*Definition IV* ^*5,3*^	
ASMI ≤ 6.34 kg/m^2^	ASMI ≤ 5.12 kg/m^2^
HGS ≤ 30 kg	HGS ≤ 20 kg
GS ≤ 0.8 m/s	GS ≤ 0.8 m/s

*Definition V* ^*5,6*^	
ASMI ≤ 6.34 kg/m^2^	ASMI ≤ 5.12 kg/m^2^
HGSv≤ 30 kg	HGS ≤ 20 kg
SPPB ≤ 8 score	SPPB ≤ 8 score

*Definition VI* ^*2,5*^	
ASMI ≤ 6.34 kg/m^2^	ASMI ≤ 5.12 kg/m^2^
HGS-BMI ≤ 24.6; <27 kg	HGS-BMI ≤ 25.1; <15.5 kg
HGS-BMI 24.7–26.3; <32 kg	HGS-BMI 25.2–28.6; <18.5 kg
HGS-BMI 26.4–28.6; <37 kg	HGS-BMI 28.7–31.6; <21.5 kg
HGS-BMI ≥ 28.7; <45 kg	HGS-BMI ≥ 31.7; <25.0 kg
Ht > 169 cm (GS <0.79 m/s)	Ht ≤ 169 cm (GS <0.76 m/s)
Ht > 169 cm (GS <0.79 m/s)	Ht ≤ 169 cm (GS <0.76 m/s)

ASMI, appendicular skeletal muscle mass index; HGS, hand grip strength; GS, gait speed; SPPB, short physical performance battery; Ht, height; BMI, body mass index. ^1^ASMI gender-specific regional cutoff points based on 2 SD below mean value for young men and women from northwest Mexico. ^2^HGS and GS based on the quintile distribution of the current study population. ^3^HGS and GS based on cutoff points based on EWGSOP 2010 criteria. ^4^ASMI, HGS, and GS gender-specific cutoff points based on EWGSOP 2010 criteria. ^5^ASMI gender-specific cutoff points based on the quintile distribution of the current study population. ^6^HGS and SPPB based on cutoff points based on EWGSOP 2010 criteria.

**Table 2:** Variation in the prevalence of sarcopenia in older Mexican adults using different cutoff points from the 2010 EWGSOP criteria.

Clinical entities	Definitions					
	I	II	III	IV	V	VI
Presarcopenia, % (*n*)	7.4 (16)	3.7 (8)	12.9 (28)	9.2 (20)	5.5 (12)	12.9 (28)
Sarcopenia, % (*n*)	1.4 (3)	4.6 (10)	16.6 (36)	7.4 (16)	7.4 (16)	13.4 (29)
Severe sarcopenia, % (*n*)	3.2 (7)	3.7 (8)	24.4 (53)	9.7 (21)	13.3 (29)	12.9 (28)
Overall prevalence of sarcopenia, % (*n*)	4.6 (10)^a^	8.3 (18)^b^	41.0 (89)^c^	17.1 (37)^d^	20.7 (45)^e^	26.3 (57)^f^

Definitions I = a, II = b, III = c, IV = d, V = e, VI = f. Values in the same row with different letters are significantly different (*p* < 0.01).

**Table 3:** Demographic, body composition, and functional characteristics of the nonsarcopenic older adults and those with sarcopenia defined by different cutoff points from the 2010 EWGSOP criteria.

Variables	Definition I		Definition II		Definition III		Definition IV		Definition V		Definition VI	
	Non-SOA (*n* = 207)	SOA (*n* = 10)	Non-SOA (*n* = 199)	SOA (*n* = 18)	Non-SOA (*n* = 128)	SOA (*n* = 89)	Non-SOA (*n* = 180)	SOA (*n* = 37)	Non-SOA (*n* = 172)	SOA (*n* = 45)	Non-SOA (*n* = 160)	SOA (*n* = 57)
Age, y	71.4 ± 6.8	79.0 ± 6.4^*∗*^	71.4 ± 6.8	76.1 ± 6.9^*∗*^	70.1 ± 6.8	74.1 ± 6.4^*∗*^	70.9 ± 6.7^*∗*^	76.1 ± 6.5^*∗*^	70.8 ± 6.7	75.4 ± 6.3^*∗*^	71.1 ± 6.8	73.5 ± 7.1^*∗*^
BW, kg	72.4 ± 13.0	55.3 ± 12.7^*∗*^	73.0 ± 12.4	55.5 ± 9.6^*∗*^	75.6 ± 11.8	65.8 ± 12.7^*∗*^	74.2 ± 11.7	58.7 ± 11.9^*∗*^	74.7 ± 11.5	59.5 ± 11.6^*∗*^	75.3 ± 11.4	60.9 ± 11.7^*∗*^
Height, m	1.6 ± 0.09	1.6 ± 0.1	1.59 ± 0.09	1.57 ± 0.1^*∗*^	1.59 ± 0.1	1.59 ± 0.1	1.59 ± 0.1	1.57 ± 0.1	1.59 ± 0.1	1.58 ± 0.1	1.59 ± 0.1	1.59 ± 0.1
BMI, kg/m^2^	28.5 ± 4.5	21.8 ± 3.3^*∗*^	28.7 ± 4.4	22.1 ± 2.9^*∗*^	29.8 ± 4.4	25.7 ± 3.8^*∗*^	29.1 ± 4.3	23.5 ± 3.0^*∗*^	29.4 ± 4.2	23.5 ± 3.0^*∗*^	29.7 ± 4.1	23.8 ± 3.2^*∗*^
FM, kg	28.6 ± 8.0	20.0 ± 6.6^*∗*^	28.8 ± 8.0	20.7 ± 5.8^*∗*^	30.2 ± 8.0	25.2 ± 7.6^*∗*^	29.4 ± 7.9	22.1 ± 6.1^*∗*^	29.9 ± 7.8	21.7 ± 6.0^*∗*^	30.2 ± 7.7	22.3 ± 6.5^*∗*^
FFM, kg	42.6 ± 8.5	34.5 ± 7.2^*∗*^	42.9 ± 8.5	34.1 ± 5.7^*∗*^	44.2 ± 8.5	39.5 ± 8.0^*∗*^	43.6 ± 8.3	35.7 ± 7.3^*∗*^	43.7 ± 8.3	36.9 ± 7.6^*∗*^	43.9 ± 8.5	37.7 ± 7.6^*∗*^
ASM, kg	15.7 ± 3.6	12.4 ± 2.7^*∗*^	15.9 ± 3.6	12.1 ± 2.2^*∗*^	16.5 ± 3.7	14.3 ± 3.2^*∗*^	16.1 ± 3.6	12.8 ± 2.9^*∗*^	16.2 ± 3.6	13.3 ± 3.1^*∗*^	16.3 ± 3.6	13.6 ± 3.1^*∗*^
ASMI, kg/m^2^	6.1 ± 0.90	4.9 ± 0.21^*∗*^	6.2 ± 0.86	4.8 ± 0.5^*∗*^	6.4 ± 0.8	5.5 ± 0.8^*∗*^	6.2 ± 0.85	5.1 ± 0.6^*∗*^	6.3 ± 0.83	5.2 ± 0.7^*∗*^	6.3 ± 0.8	5.3 ± 0.71^*∗*^
FMI, kg/m^2^	16.0 ± 4.3	14.5 ± 3.0	16.0 ± 4.4	15.1 ± 3.2	16.3 ± 4.5	15.4 ± 3.9	16.0 ± 4.4	15.6.7 ± 3.4	16.2 ± 4.4	14.9 ± 3.7	16.4 ± 4.3	14.8 ± 3.8^*∗*^
WC, cm	96.9 ± 11.5	85.1 ± 13.8^*∗*^	97.6 ± 11.1	82.9 ± 11.6^*∗*^	98.9 ± 10.7	92.6 ± 12.5^*∗*^	98.3 ± 10.8	86.9 ± 11.9^*∗*^	98.9 ± 10.6	86.8 ± 11.5^*∗*^	99.5 ± 10.4	87.7 ± 11.5^*∗*^
BMC, kg	1.9 ± 0.47	1.7 ± 0.43	1.98 ± 0.5	1.6 ± 0.4^*∗*^	2.0 ± 0.5	1.9 ± 0.5^*∗*^	2.0 ± 0.5	1.7 ± 0.5^*∗*^	2.0 ± 0.46	1.8 ± 0.5^*∗*^	2.00 ± 0.5	1.82 ± 0.5^*∗*^
HGS, kg	23.7 ± 8.6	17.7 ± 6.3^*∗*^	23.9 ± 8.6	18.3 ± 5.2^*∗*^	24.6 ± 9.4	21.8 ± 6.9^*∗*^	24.4 ± 8.8	19.1 ± 5.1^*∗*^	24.1 ± 8.8	21.1 ± 6.9^*∗*^	23.7 ± 8.9	22.7 ± 7.4
GS, m/s	0.9 ± 0.2	0.8 ± 0.2	0.9 ± 0.2	0.9 ± 0.2	0.9 ± 0.2	0.8 ± 0.2	0.9 ± 0.2	0.8 ± 0.2	0.9 ± 0.2	0.8 ± 0.2	0.9 ± 0.2	0.9 ± 0.2
SPPB, score	8.0 ± 1.7	7.4 ± 1.6	8.0 ± 1.7	7.8 ± 1.0	8.3 ± 1.9	7.5 ± 1.3^*∗*^	8.1 ± 1.7	7.4 ± 1.4^*∗*^	8.1 ± 1.8	7.4 ± 1.3^*∗*^	8.0 ± 1.8	7.9 ± 1.6
ADL, %	1.9	0.0	2.0	0.0	1.6	2.3	2.2	0.0	2.3	0.0	2.5	0.0
IADL, %	0.5	0.0	0.5	0.0	0.0	1.1	0.6	0.0	0.6	0.0	0.6	0.0
Gender, %												
Men	65.7	60.0	35.2	27.8	69.5	59.5	34.4	35.1	32.6	42.2	31.2	43.9
Women	34.3	40.0	64.8	72.2	30.5	40.5	65.6	64.9	67.4	57.8	68.8	56.1
SES, %												
High	13.5	10.0	13.6	11.1	13.3	13.5	13.9	10.8	13.4	13.3	13.1	14.0
Medium	49.3	30.0	49.8	33.3	49.2	47.2	49.4	43.2	50.0	44.4	35.6	45.6
Low	37.2	60.0	36.7	55.6	37.5	39.3	36.7	46.0	36.6	44.4	35.6	45.6
Schooling, %												
HS	50.2	50.0	49.8	55.6	53.1	46.1	51.1	46.0	50.6	48.9	48.8	54.4
HS or less	49.8	50.0	50.2	44.4	46.9	53.9	48.9	54.0	49.4	51.1	51.2	45.6

Non-SOA, nonsarcopenic older adults; SOA, sarcopenic older adults; BW, body weight; BMI, body mass index; FM, fat mass; FFM, fat-free mass; ASM, appendicular skeletal muscle mass; ASMI, appendicular skeletal muscle mass index; FMI, fat mass index; WC, waist circumference; BMC, bone mineral content; HGS, hand grip strength; GS, gait speed; SPPB, short physical performance battery; ADL, activities of daily living; IADL, instrumental activities of daily living; SES, socioeconomic status; HS, high school.

**Table 4:** Health and nutritional characteristics of the nonsarcopenic older adults and those with sarcopenia defined by different cutoff points from the 2010 EWGSOP criteria.

Variables	Definition I		Definition II		Definition III		Definition IV		Definition V		Definition VI	
	Non-SOA (*n* = 207)	SOA (*n* = 10)	Non-SOA (*n* = 199)	SOA (*n* = 18)	Non-SOA (*n* = 128)	SOA (*n* = 89)	Non-SOA (*n* = 180)	SOA (*n* = 37)	Non-SOA (*n* = 172)	SOA (*n* = 45)	Non-SOA (*n* = 160)	SOA (*n* = 57)
Low BW, %												
Yes (T1)	12.6	50.0^*∗*^	10.6	55.6^*∗*^	5.5	27.0^*∗*^	6.7	51.4^*∗*^	5.2	48.9^*∗*^	^*∗*^4.4	42.1^*∗*^
No (T2 and T3)	87.4	50.0	89.4	44.4	94.5	73.0	93.3	48.6	94.8	51.1	95.6	57.9
Low FM, %												
Yes (T1)	14.0	50.0^*∗*^	13.1	44.4^*∗*^	10.2	23.6^*∗*^	12.8	29.7^*∗*^	11.6	31.1^*∗*^	10.6	29.8^*∗*^
No (T2 and T3)	86.0	50.0	86.9	55.6	89.8	76.4	87.2	70.3	88.4	68.9	89.4	70.2
Low FFM, %												
Yes (T1)	11.1	60.0^*∗*^	9.1	61.1^*∗*^	3.9	27.0^*∗*^	5.6	51.4^*∗*^	4.7	46.7^*∗*^	4.4	38.6^*∗*^
No (T2 and T3)	88.9	40.0	99.9	38.9	96.1	73.0	94.4	48.6	95.3	53.3	95.6	61.4
Comorbidity, %												
Mild/moderate	78.9	80.0	75.4	83.3	77.3	74.2	77.2	70.3	77.3	71.1	76.9	73.7
High	24.1	20.0	24.6	16.7	22.7	25.8	22.8	29.7	22.7	28.9	23.1	26.3
CDys, %												
Yes	48.3	40.0	48.7	38.9	53.1	40.5	48.9	43.2	50.6	37.8	52.5	35.1^*∗*^
No	51.7	60.0	51.3	61.1	46.9	59.5	51.1	56.8	49.4	62.2	47.5	64.9
HBP, %												
Yes	41.8	20.0	42.9	16.7^*∗*^	45.3	34.1	41.3	37.8	42.7	33.3	44.0	31.6
No	58.2	80.0	57.1	83.3	54.7	65.9	58.7	62.2	57.3	66.7	56.0	69.3
Osteoporosis, %												
Yes	15.9	20.0	14.6	33.3^*∗*^	10.9	23.6^*∗*^	13.9	27.0^*∗*^	14.0	24.4	13.8	22.8
No	84.1	80.0	85.4	66.7	89.1	76.4	86.1	73.0	86.0	75.6	86.2	77.2
Polypharmacy, %												
Yes	34.3	20.0	34.2	27.8	33.6	33.7	34.4	29.7	34.9	28.9	35.6	28.1
No	65.7	80.0	65.8	72.2	66.4	66.3	65.6	70.3	65.1	71.1	64.4	71.9
Smoking, %												
Yes	37.6	50.0	37.6	44.4	34.7	43.2	36.5	46.0	36.5	44.4	36.6	42.1
No	62.4	50.0	62.4	55.6	65.3	56.8	63.5	54.0	63.5	55.6	63.3	57.9
AC, %												
Yes	36.7	10.0	36.7	22.2	35.2	36.0	38.3	21.6^*∗*^	36.6	31.1	35.6	35.1
No	63.3	90.0	63.3	77.8	64.8	64.0	61.7	78.4	63.4	68.9	64.4	64.9
PAL, %												
Active/moderately	5.3	20.0^*∗*^	5.0	16.7^*∗*^	3.1	10.1^*∗*^	5.0	10.8	4.1	13.3^*∗*^	3.1	14.0^*∗*^
Sedentary/light	94.7	80.0	95.0	83.3	96.9	89.9	95.0	89.2	95.9	86.7	96.9	86.0

Non-SOA, nonsarcopenic older adults; SOA, sarcopenic older adults; BW, body weight; FM, fat mass; FFM, fat-free mass; T, tertile; CDys, cognitive dysfunction; AC, alcohol consumption; HBP, high blood pressure; PAL, physical activity level.

## Data Availability

Access to data is restricted. For ethical concerns and privacy of the volunteers, the access to data is on request.

## Supplementary Materials

Supplementary Materials Supplementary Table 1: the results of the univariate analysis using several risk variables for sarcopenia. For this analysis, sarcopenia diagnosed by definition I was considered. The significant association was considered a *p* value of ≤0.2. Supplementary Table 2: the final, gender-adjusted model for the association between some factors and sarcopenia. Model 1 shows that the risk of sarcopenia was 1.16 times greater for each yearly increase in age (CI 1.04–1.29; *p* = 0.006) and the risk sarcopenia was 11.08 times greater for low FFM category (CI 2.71–45.24; *p* = 0.001).
