# Supplementary material for: Sarcopenia: Influence of Regional Skeletal Muscle Cutoff Points and Fat-Free Mass in Older Mexican People—A Pilot Study
Source: Curr Gerontol Geriatr Res. 2020 May 31;2020:8037503. doi: 10.1155/2020/8037503 (PMC7281843; doi:10.1155/2020/8037503)
Supplement: Supplementary Materials — Supplementary Table 1: the results of the univariate analysis using several risk variables for sarcopenia. For this analysis, sarcopenia diagnosed by definition I was considered. The significant association was considered a p value of ≤0.2. Supplementary Table 2: the final, gender-adjusted model for the association between some factors and sarcopenia. Model 1 shows that the risk of sarcopenia was 1.16 times greater for each yearly increase in age (CI 1.04–1.29; p = 0.006) and the risk sarcopenia was 11.08 times greater for low FFM category (CI 2.71–45.24; p = 0.001). [file 8037503.f1.docx]

With respect to the main association between sarcopenia and several risk factors examined. Results of the univariate analysis using sarcopenia diagnosed by definition I showed a p value of ≤0.2 for age, FM, BMI, FFM, ASM, ASMI, WC, BMC, HGS, GS, PAL, low body weight, low FM and low FFM, high blood pressure and alcohol consumption **(Supplementary Table 1).** All these variables (except those that form part of the definitions; *i.e.*, ASMI, HGS, SPPB and GS) were then entered into a stepwise multivariate regression analysis. **Supplementary Table 2** shows the final, gender-adjusted model for the association between some factors and sarcopenia. Model 1 shows that the risk of sarcopenia was 1.16 times greater for each yearly increase in age (CI 1.04-1.29; p = 0.006) and the risk sarcopenia was 11.08 times greater for low FFM category (CI 2.71-45.24; p = 0.001). Based on these results, futures studies in local contexts should consider other nutritional parameters as markers of undernutrition. Recently, it was published that low BMI, decreased mini-nutritional assessment score, and low serum albumin level were associated with major risk of sarcopenia in Iranian older adults [43].

**Supplementary Table 2.** Results of stepwise multiple regression analyses for sarcopenia defined by definition I.

| **Independent variables** | **Model** | |
| --- | --- | --- |
|  | OR (CI 95%) | *p*-Value |
| Age, years | 1.16 (1.04-1.29) | 0.006 |
| Low FFM, %  No (T2 and T3)  Yes (T1) | Ref  11.08 (2.71-45.24) | 0.001 |

FFM, Fat-free mass; T, Tertile; OR, Odds ratio; CI, Confidence intervals. Stepwise by logistic regression analysis using sarcopenia defined by criteria I as the dependent variable. Model included gender, age and FFM.
